# Influence of renal function on the ability of TyG Index to predict all-cause mortality

**DOI:** 10.1186/s12944-023-01958-1

**Published:** 2023-11-11

**Authors:** Huilan Li, Weihua Chen, Xueqin Lin, Weiqin Chen, Tingzheng Xie, Kaihong Chen, Shuhong Hou, Huaqing Li

**Affiliations:** 1 Longyan First Affiliated Hospital of Fujian Medical University, Longyan, 364000 China; 2 Beijing Friendship Hospital, Capital Medical University, Beijing, 100053 China; 3 School of Basic Medical Sciences, Fujian Medical University, Fuzhou, 350000 China

**Keywords:** Triglyceride-glucose index, Insulin resistance, Renal function, All-cause mortality

## Abstract

**Background:**

The association between triglyceride–glucose (TyG) index and poor prognosis remains controversial. Whether renal function status affects the ability of the TyG index to predict poor prognosis has not yet been elucidated and merits further studies.

**Methods:**

This retrospective cohort study included 22,031 participants from communities in the U.S. By juxtaposing the TyG categories with the estimated glomerular filtration rate (eGFR, either < 60 mL/min/1.73m^2^ or ≥ 60 mL/min/1.73m^2^), participants were categorized into four distinct groups: (1) TyG_L/eGFR_H; (2) TyG_H/eGFR_H; (3) TyG_L/eGFR_L; and (4) TyG_H/eGFR_L. The endpoint was the all-cause mortality rate. Standard Kaplan–Meier plots were constructed and multifactor Cox regression analyses were carried out and restricted cubic spline regression analysis was utilized to assess the association between death and the TyG index for different renal function statuses.

**Results:**

No statistical differences were found in the TyG groups in participants with normal renal function after adjustment for all covariates (*P* = 0.070). However, in the high TyG index group with renal insufficiency, the risk of all-cause mortality rates was reduced by 18%. (HR, 0.82; CI, 0.69–0.98). The TyG index (high vs. low) and renal function (eGFR < 60 vs. eGFR ≥ 60) had statistically significant interactions with death (P < 0.001). When all covariates were adjusted, the risk of mortality for the TyG_L combined with eGFR_L group was 56% higher than that for the TyG_L combined with eGFR_H group (HR, 1.56; CI, 1.33–1.82). In the renal insufficiency population, a nonlinear relationship was observed between mortality and the TyG index, albeit with a differing pattern (*P* for nonlinearity < 0.001).

**Conclusions:**

While it has been known that TyG index was a prognosis marker of CVD, this research highlights that higher TyG index was associated with higher all-cause mortality rates for all participants. Furthermore, renal function status significantly moderates the effect of the TyG index on all-cause mortality in community-dwelling adults.

**Supplementary Information:**

The online version contains supplementary material available at 10.1186/s12944-023-01958-1.

## Background

Insulin resistance (IR), typically characterized by the diminished responsiveness of target organs to circulating insulin, is a well-acknowledged marker for both metabolic disorders and systemic inflammation [1, 2]. Recently, the triglyceride-glucose (TyG) index, calculated as the logarithmic product of fasting triglyceride (TG) and glucose concentrations, has emerged as a potential alternative to IR and was superior to HOMA-IR [3, 4].

Over recent years, TyG index has gained attention due to its potential in predicting clinical outcomes like atrial fibrillation, cardiovascular disease (CVD), and stroke [5–7]. Although the association between poor prognosis and a high TyG index has been reported in most contemporary studies [8], there remains a notable discrepancy in the literature. Some studies failed to cement a clear connection between it and prognosis [9–11], and others even showed an absence of significant correlation [12]. This divergence in findings raised questions about potential external factors that might influence TyG index’s predictive capacity. Perhaps there are underlying conditions or states that modulate its accuracy [13].

With these queries in mind, our attention was drawn to a recent study that documented the dysregulation of renal insulin receptors. This dysregulation not only impacts local renal metabolism but also disrupts systemic glucose homeostasis, leading to systemic metabolic abnormalities [14]. This revelation suggested a potential intertwined relationship among IR, renal insufficiency, and poor prognosis. Given that both insulin resistance and renal insufficiencies are pervasive conditions with well-established links to adverse clinical outcomes, it became imperative to investigate their potential influence on TyG index’s prognostic capability.

Thus, arising from these concerns and the highlighted knowledge gap in the interplay between renal function and the TyG index, this research aimed to determine whether the accuracy of using the TyG as a predictor of poor prognosis is altered by the renal function status.

## Methods

### Study design and participants

The National Health and Nutrition Examination Survey (NHANES) is a national survey, which initiative of the U.S. National Center for Health Statistics (NCHS). It employs a stratified multistage random sampling strategy, with data being released in biennial cycles starting from 1999. After obtaining informed consent, selected participants complete a personal questionnaire at home, which is followed by physical and laboratory examinations as well as a 24-h dietary recall conducted at a mobile screening center. More comprehensive details about this survey are available on the NHANES website. And a retrospective analysis of publicly accessible NHANES data was performed in the present study.

**Fig. 1:**
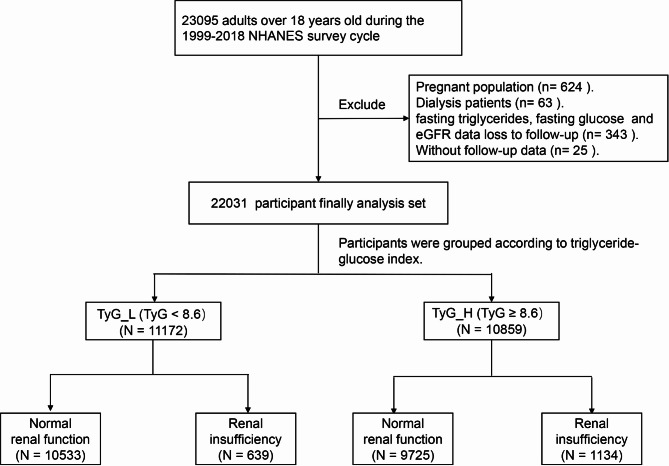
Flow diagram depicting the study flow

In this study, participants ≥ 18 years of age from U.S. communities were included. Of these, participants for whom fasting triglycerides, glucose, or estimated glomerular filtration rate (eGFR) data were not available were excluded. Also excluded are those who were Pregnant women, patients undergoing dialysis, and 25 participants who were not followed up. Consequently, 22031 individuals were enrolled in this study (Fig. 1).

### Exposure

In this research, the TyG index was the exposure of interest, which was calculated at the time of study inclusion based on parameters measured while fasting. This index was specific calculations based on the formula of Luis E et al.: TyG index = Ln [1/2(TG (mg/dL) × FPG (mg/dL)] [4]. Blood samples for measuring plasma glucose and triglycerides were exclusively taken in the morning. We ensured that these measurements were obtained only from participants aged 12 and above who had fasted for a minimum of 8 h and not exceeding 24 h. Renal function, represented by eGFR, was calculated from serum creatinine levels using the Chronic Kidney Disease (CKD) Epidemiology Collaborative’s creatinine equation as a means to establish the baseline CKD status, participants were considered to have CKD if their eGFR was less than 60 mL/min/1.73m^2^ or if their Urinary Albumin to Creatinine Ratio was greater than or equal to 30 mg/g [15]. A restricted cubic spline function analysis determined the optimal TyG index threshold was to be 8.6, which led to the categorization of the participants into two groups: the TyG_L group with a TyG index of < 8.6, and the TyG_H group with a TyG index of ≥ 8.6. By juxtaposing the TyG categories with eGFR (either < 60 mL/min/1.73m^2^ or ≥ 60 mL/min/1.73m^2^), four distinct groups were obtained: (1) TyG_L/eGFR_H; (2) TyG_H/eGFR_H; (3) TyG_L/eGFR_L; and (4) TyG_H/eGFR_L.

### Definition of variables of interest

For this study, the following factors were selected as covariates: the confounding factors of gender (male or female), age, education level (less than high school diploma, high school diploma, college or university degree or above), and race (Mexican American, non-Hispanic black, non-Hispanic white, other Hispanic, and other races) were used as confounders related to the demographic characteristics. The factors related to lifestyle included smoking (former or current smoker) and drinking habits. Body mass index (BMI) was measured by using the formula of measured weight in kilograms (kg) divided by the height squared (m^2^). The clinical comorbidities included hypertension (defined as having a physician diagnosed history of hypertension or a history of use of antihypertensive medication, the measured mean systolic blood pressure ≥ 140 mmHg, the measured mean diastolic blood pressure ≥ 90 mmHg), diabetes mellitus (defined as a history of physician-diagnosed diabetes mellitus or the use of antihyperglycemic medication, measured fasting blood glucose ≥ 7.0 mmol/L, measured glycosylated hemoglobin [HbA1c] ≥ 6.5%), hyperlipidemia (yes/no), and a history of cardiovascular disease defined as self-reported diagnosis by a physician of congestive heart failure, coronary heart disease, angina pectoris, heart attack, or stroke. Information on the levels of fasting triglycerides (mg/dL), LDL-C (mg/dL), HDL-C (mg/dL), blood pressure (mmHg), and fasting blood glucose (mg/dL) were determined at the time of the medical center visit. The categories of chronic kidney disease were defined in accordance with the KDIGO (Kidney Disease: Improving Global Outcomes) guidelines. Detailed information about the data on potential confounders were available on the NCHS website.

### Mortality outcomes

All-cause mortality during follow-up was the outcome of this study. The International Statistical Classification of Diseases, 10th edition, was used as the basis for categorizing all causes of death in this study. Mortality follow-up data for NHANES participants from 1999 to 2018 were available until December 31, 2019.

### Statistical analyses

The weights suggested by NHANES were used in the analysis of this study. Continuous variables were subjected to ANOVA testing and were reported as mean standard error (SE). Assessment of categorical variables was assessed through the use of the chi-squared test.

To assess the relationship between death and the TyG index and to further determine whether TyG was differentially predictive of mortality in individuals with different renal function status, standard Kaplan–Meier plots and a single multifactor Cox regression analysis were used. Sensitivity analysis of cardiovascular and non-cardiovascular mortality risk according to the TyG index and different renal functions using multifactorial Cox regression analysis. Model 1 was a basic design that was not adjusted for potential confounders. On the contrary, Model 2 included adjustments for age, sex, and race, and potential confounders were fully taken into account in Model 3, such as alcohol consumption, smoking states, BMI, education level, HDL cholesterol level, LDL cholesterol level, eGFR, hypertension, DM, and CVD. To investigate the nonlinear relationship between mortality and TyG in the general population and in those with different renal function statuses, restricted cubic spline regression analysis was performed.

A two-sided p-value of less than 0.05 was defined as statistically significant in this study. The R programming environment (version 4.2.2; Vienna, Austria) was used for data analysis.

## Results

The TyG index was used to separate the research participants based on their basic characteristics, as shown in Table 1. Participants in the TyG_H group were typically elder, overweight, and apt to the male sex compared with the TyG_L group (all *P* < 0.001). Based on the CKD G category results, the TyG_H group displayed a higher prevalence of CKD, with 36.0% in stage 2, 2.9% in stage 3a, 2.0% in stage 3b, 0.6% in stage 4, and 0.4% in stage 5. Furthermore, participants in the TyG_H group exhibited higher systolic and diastolic blood pressure, FPG, LDL-C, and TG levels; smoking prevalence; and incidence of DM, hypertension, and CVD (all *P* < 0.001).

**Table 1:** Baseline data according to the level of TyG

Characteristics	TyG_L (TyG < 8.5) N = 11172	TyG_H (TyG ≥ 8.5) N = 10859	P-value	
**Age(year)**	43.4 ± 0.3	50.5 ± 0.3	< 0.001	
**Female, n (%)**	6183(56.6)	4943(44.9)	< 0.001	
**Race, n (%)**			< 0.001	
Mexican American	1652(7.2)	2341(9.3)		
Non-Hispanic Black	2966(14.8)	1380(7.0)		
Non-Hispanic White	4625(66.0)	5162(71.0)		
Other Hispanic	856(5.3)	1020(5.9)		
Other Race	1073(6.8)	956(7.0)		
**Education level, n (%)**				
< High school	2362(14. 8)	3320(20.4)	< 0.001	
High school	3597(37.7)	3621(39.3)		
≥ High school	4588(47.5)	3733(40.3)		
**Smoke status, n (%)**				
Former smoker	2305(22.1)	3099(28.8)	< 0.001	
Current smoker	2053(19.7)	2307(22.7)		
**Alcohol using, n (%)**	8326(88.8)	8450(88.7)	< 0.001	
**BMI (kg/m**^**2**^)	27.0 ± 0.1	30.6 ± 0.1		
**SBP (mmHg)**	118.2 ± 0.2	125.0 ± 0.2		
**DBP (mmHg)**	68.9 ± 0.2	72.1 ± 0.2		
**LDL-C (mg/dl)**	109.0 ± 0.5	122.8 ± 0.5	< 0.001	
**HDL-C (mg/dl)**	59.5 ± 0.3	47.0 ± 0.2	< 0.001	
**Fasting glucose (mg/dl)**	96.2 ±0.2	114.3 ±0.5	< 0.001	
**Fasting TG (mg/dl)**	73.9 ±0.3	191.8 ±1.9	< 0.001	
**eGFR (mL/min/1.73 m**^**2**^)	98.8 ±0.4	91.7 ±0.4	< 0.001	
**CKD G category, n (%)**				
1	7530(67.3)	5908(56.0)	< 0.001	
2	3003(28.6)	3817(36.0)		
3a	1162(5.3)	429(2.9)		
3b	159(0.9)	295(2.0)		
4	42(0.2)	95(0.6)		
5	9(0.0)	11(0.1)		
**Family DM, n (%)**	4174(36.8)	5069(46.3)	< 0.001	
**Family CVD, n (%)**	1157(12.0)	1474(15.1)	< 0.001	
**Hypertension, n (%)**	4678(36.8)	6829(60.6)	< 0.001	
**DM, n (%)**	853(5.3)	3205(23.5)	< 0.001	
**Hyperlipidemia, n (%)**	6050(53.1)	9858(91.1)	< 0.001	
**CVD, n (%)**	859(5.9)	1515(11.8)	< 0.001	

TyG, triglyceride-glucose; BMI, Body mass index; SBP, Systolic blood pressure; DBP, Diastolic blood pressure; FPG, Fasting plasma glucose; HDL-C, high-density lipoprotein cholesterol; LDL-C, Low-density lipoprotein-C; TG, triglyceride; eGFR: estimated glomerular filtration rate; CVD, Cardiovascular Disease; DM, diabetes mellitus

The study population was further categorized according to the TyG index and eGFR (eGFR_L vs. eGFR_H). Table 2 demonstrates that the TyG_H combined with the eGFR_L group had the highest proportion of older patients and those with comorbidities, such as DM, hypertension, hyperlipidemia, and CVD (all *P* < 0.001).

**Table 2:** Baseline according to the level of TyG according to different kidney function

Characteristics	eGFR ≥ 60			eGFR < 60		P-value	
	TyG_L N = 10533	TyG_H N = 9725		TyG_L N = 639	TyG_H N = 1134		
**Age(year)**	42.1 ± 0.3	48.6 ± 0.2		74.0 ± 0.5	72.4 ± 0.5	< 0.001	
**Female, n (%)**	5852(56.4)	4341(43.7)		331(60.0)	602(58.3)	< 0.001	
**Race, n (%)**							
Mexican American	1622(7.5)	2237(10.0)		30(2.0)	104(3.0)	< 0.001	
Non-Hispanic Black	2811(14.8)	1235(6.9)		155(13.5)	145(7.3)		
Non-Hispanic White	4238(65.5)	4392(69.9)		387(76.3)	770(83.2)		
Other Hispanic	826(5.4)	958(6.1)		30(3.3)	62(2.9)		
Other Race	1036(6.8)	903(7.2)		37(5.0)	53(3.7)		
**Education level, n (%)**							
< High school	2174(14.4)	2944(20.0)		188(23.5)	376(25.0)	< 0.001	
High school	3366(37.9)	3216(39.3)		231(34.6)	405(39.9)		
≥ High school	4369(47.7)	3384(40.8)		219(42.0)	349(35.1)		
**Smoke status, n (%)**							
Former smoker	2055(21.4)	2641(27.9)		250(40.1)	458(39.5)	< 0.001	
Current smoker	1994(20.2)	2199(23.8)		59(8.2)	108(10.0)		
**Alcohol using, n (%)**	7856(89.0)	7650(89.6)		470(83.2)	800(78.7)	< 0.001	
**BMI (kg/m**^**2**^)	27.0 ± 0.1	30.6 ± 0.1		27.8 ± 0.3	30.3 ± 0.2		
**SBP (mmHg)**	117.5 ± 0.2	124.2 ± 0.2		133.5 ± 1.2	134.8 ± 0.9		
**DBP (mmHg)**	69.1 ± 0.2	72.7 ± 0.2		64.3 ± 0.7	65.5 ± 0.5		
**LDL-C (mg/dl)**	109.3 ± 0.5	123.8 ± 0.5		101.8 ± 2.1	112.1 ± 1.4	< 0.001	
**HDL-C (mg/dl)**	59.4 ± 0.3	46.9 ± 0.2		62.8 ± 1.0	48.7 ± 0.5	< 0.001	
**Fasting glucose (mg/dl)**	96.0 ± 0.2	113.5 ± 0.5		101.8 ± 1.0	123.3 ± 1.7	< 0.001	
**Fasting TG (mg/dl)**	73.7 ± 0.4	192.9 ± 2.0		77.1 ± 1.3	179.6 ± 3.3	< 0.001	
**eGFR (mL/min/1.73 m**^**2**^)	100.9 ± 0.3	95.5 ± 0.3		48.8 ± 0.4	47.4 ± 0.4	< 0.001	
**Family DM, n (%)**	3944(37.0)	4552(46.3)		230(32.5)	517(46.3)	< 0.001	
**Family CVD, n (%)**	1082(11.5)	1319(15.2)		75(16.0)	155(14.3)	< 0.001	
**Hypertension, n (%)**	4124(34.8)	5842(58.2)		554(84.7)	987(87.9)	< 0.001	
**DM, n (%)**	691(4.6)	2667(21.7)		162(23.1)	538(44.3)	< 0.001	
**Hyperlipidemia, n (%)**	5583(52.2)	8812(91.0)		467(75.5)	1046(92.9)	< 0.001	
**CVD, n (%)**	624(4.7)	1082(9.6)		235(34.7)	433(37.2)	< 0.001	

TyG, triglyceride-glucose; BMI, Body mass index; SBP, Systolic blood pressure; DBP, Diastolic blood pressure; FPG, Fasting plasma glucose; HDL-C, high-density lipoprotein cholesterol; LDL-C, Low-density lipoprotein-C; TG, triglyceride; eGFR: estimated glomerular filtration rate; CVD, Cardiovascular Disease; DM, diabetes mellitus

**Fig. 2:**
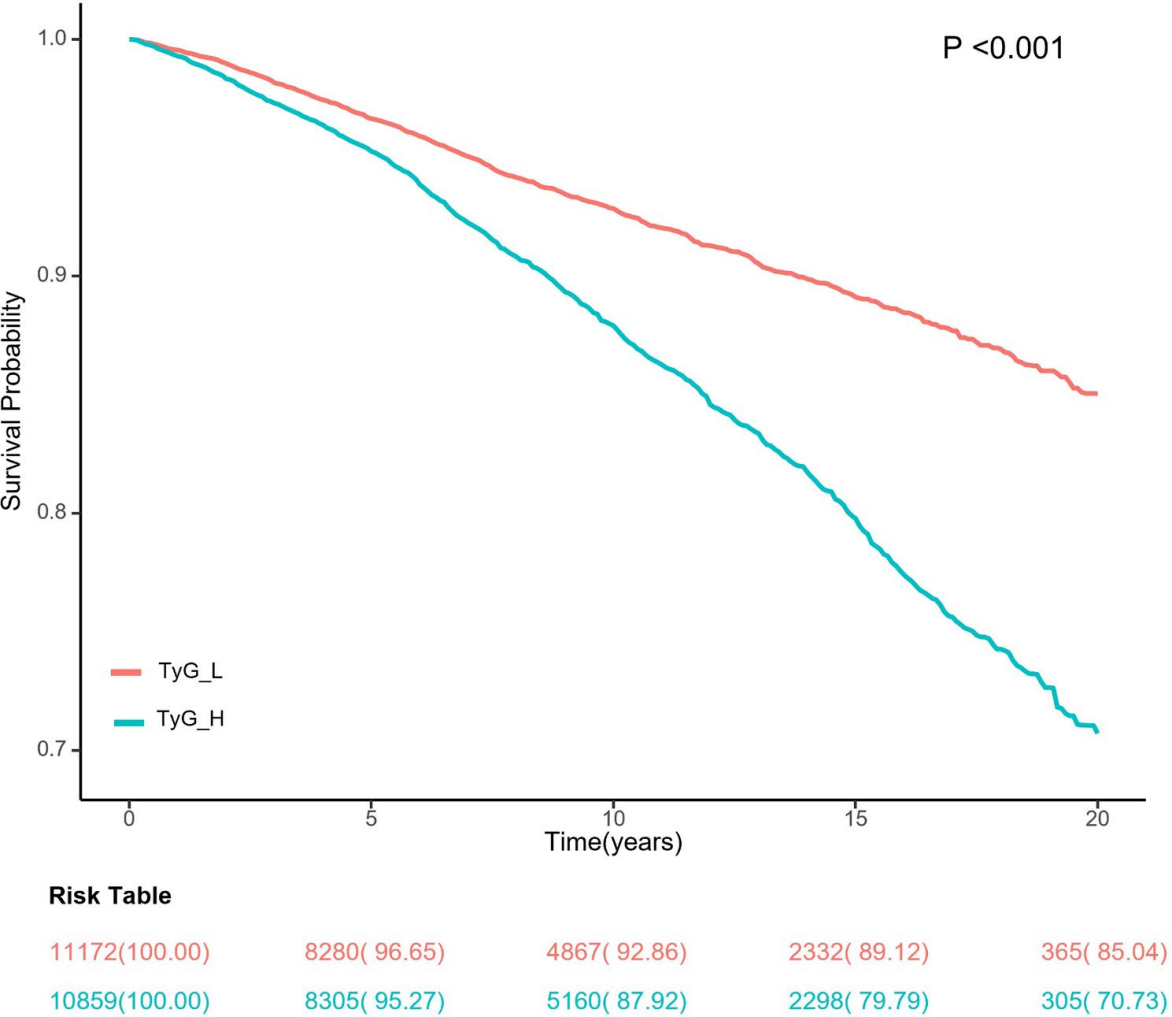
Kaplan‒Meier curve analysis for all-cause mortality and the TyG index

Kaplan–Meier curves indicated a powerful connection between death and the TyG index in our research participants (all *P* < 0. 001; Fig. 2). When all covariates were adjusted, a high TyG index corresponded to a 53% increased risk of mortality (*P* = 0.047). Using Cox proportional risk analysis, it revealed that the TyG index increased by 1 unit was associated with a 13% increase in mortality risk (*P* = 0.010; Table 3).

**Table 3:** The HR (95% CI) of All-cause mortality with TyG, and according to different renal function from the three models

Characteristics	Model 1			Model 2			Model 3		P for interaction
	HR (95% CI)	P-value		HR (95% CI)	P-value		HR (95% CI)	P-value	
**TyG(per 1 unit)**	1.65 (1.55, 1.75)	< 0.001		1.27 (1.18, 1.36)	< 0.001		1.13 (1.02, 1.25)	0.010	
**TyG_group**									
TyG_L	Reference			Reference			Reference		
TyG_H	1.89 (1.74,2.05)	< 0.001		1.19 (1.10,1.28)	< 0.001		1.04 (0.94,1.14)	0.470	
**Subgroup**									
**eGFR ≥ 60**									< 0.001
TyG(per 1 unit)	1.65 (1.53, 1.77)	< 0.001		1.28 (1.18, 1.39)	< 0.001		1.18 (1.05, 1.33)	0.004	
TyG_L	Reference			Reference			Reference		
TyG_H	1.93 (1.74,2.14)	< 0.001		1.25 (1.13,1.37)	< 0.001		1.11 (0.99,1.25)	0.070	
**eGFR < 60**									
TyG(per 1 unit)	1.04 (0.90, 1.20)	0.620		1.18 (1.02, 1.37)	0.030		1.00 (0.82, 1.22)	0.980	
TyG_L	Reference			Reference			Reference		
TyG_H	0.80 (0.67,0.96)	0.010		0.92 (0.81,1.05)	0.240		0.82 (0.69,0.98)	0.030	

Model 1: Not adjusted. Model 2: Adjusted for age, sex and race. Model 3: Adjusted for age, sex, race, education level, alcohol consumption, smoking status, BMI, LDL-C level, HDL-C level, eGFR, hypertension, DM and CVD.

CI, Confidence interval; TyG, Triglyceride-glucose index; HR, Hazard ratio

Using univariate Cox proportional hazard analysis, it showed that the TyG index increased by 1 unit was associated with an 18% increase in mortality risk (*P* = 0.004) for participants with normal renal function. However, no statistically significant increase in the mortality risk was observed for individuals with renal insufficiency (*P* = 0.980). When all covariates were considered, no significant difference was noted among the TyG groups in participants with normal renal function (*P* = 0.070); however, the high TyG index group with renal insufficiency had an 18% reduced risk of death (HR, 0.82; CI, 0.69–0.98; Table 3). The TyG index (high vs. low) and renal function (eGFR < 60 vs. eGFR ≥ 60) had statistically significant interactions with all-cause mortality (*P* < 0.001).

**Fig. 3:**
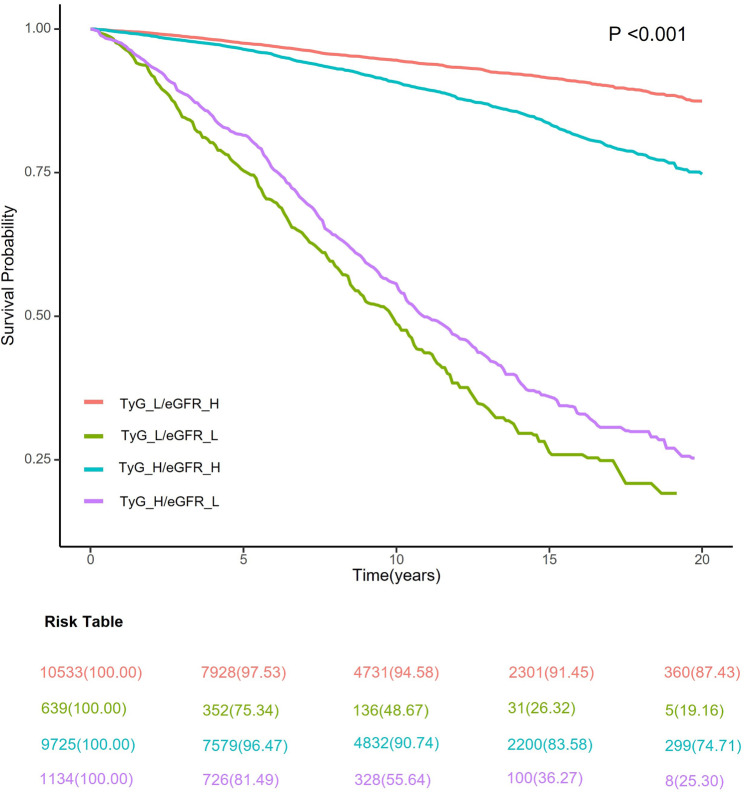
Kaplan‒Meier curve analysis for all-cause mortality according to the TyG index and renal function status

Further categorization of the participants based on renal function status revealed distinct all-cause mortality trends within the TyG groups (all *P* < 0.001; Fig. 3). In the analysis of all-cause mortality, a significant difference was not perceived between the TyG_H combined with the eGFR_H group and the TyG_L combined with the eGFR_H group (*P* = 0.090) after adjusting for all covariates. However, the risk of mortality was 56% higher in the TyG_L combined with the eGFR_L group compared with the TyG_L combined with the eGFR_H group (HR, 1.56; CI, 1.33–1.82; Table 4). When we further disaggregated the causes of death, we found that the relationship between the TyG and the risk of cardiovascular or non-cardiovascular mortality in adults with renal insufficiency was generally consistent with the results for all-cause mortality (Supplementary Table 1).

**Table 4:** The HR (95% CI) of All-cause mortality according to TyG and different renal function from the three models

Characteristics	Model 1			Model 2			Model 3	
	HR (95% CI)	P-value		HR (95% CI)	P-value		HR (95% CI)	P-value
TyG_L/eGFR_H	Reference			Reference			Reference	
TyG_L/eGFR_L	13.56 (11.30, 16.26)	< 0.001		1.79(1.56, 2.05)	< 0.001		1.56 (1.33, 1.82)	< 0.001
TyG_H/eGFR_H	1.93 (1.74, 2.14)	< 0.001		1.25(1.13, 1.37)	< 0.001		1.10 (0.98, 1.23)	0.090
TyG_H/eGFR_L	10.85 (9.32, 12.63)	< 0.001		1.71(1.49, 1.96)	< 0.001		1.36 (1.16, 1.60)	< 0.001

Model 1: Not adjusted. Model 2: Adjusted for age, sex and race. Model 3: Adjusted for age, sex, race, education level, alcohol consumption, smoking status, BMI, LDL-C level, HDL-C level, eGFR, hypertension, DM and CVD.

CI, Confidence interval; TyG, Triglyceride-glucose index; HR, Hazard ratio

**Fig. 4:**
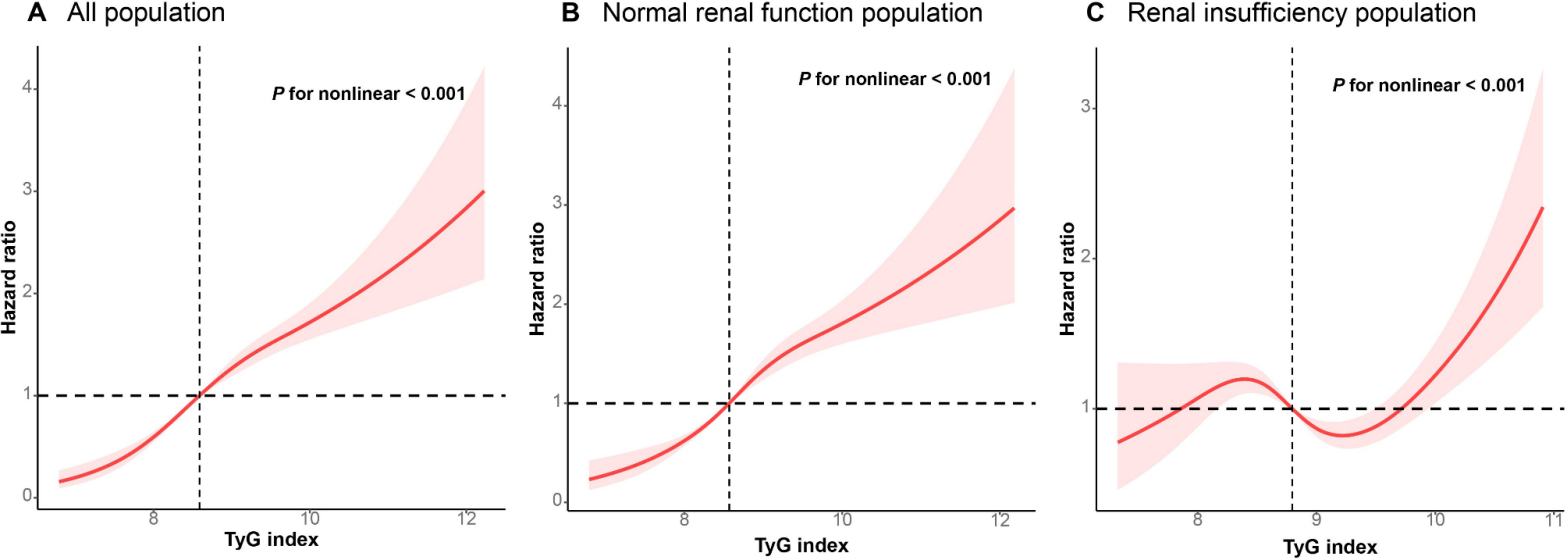
Hazard ratios for all-cause mortality based on restricted cubic spine functions for the TyG index in people with normal renal functions (**A, B**); the TyG index and all-cause mortality in patients with renal insufficiency (**C**)

The results of restricted cubic spline regression analysis alluded to a nonlinear relationship between mortality and TyG in the general population and in those with normal renal function (*P* for nonlinearity < 0.001; Fig. 4A and B). A similar nonlinear trend was also noted in individuals with renal insufficiency, albeit with a differing pattern (*P* for nonlinearity < 0.001; Fig. 4C).

**Fig. 5:**
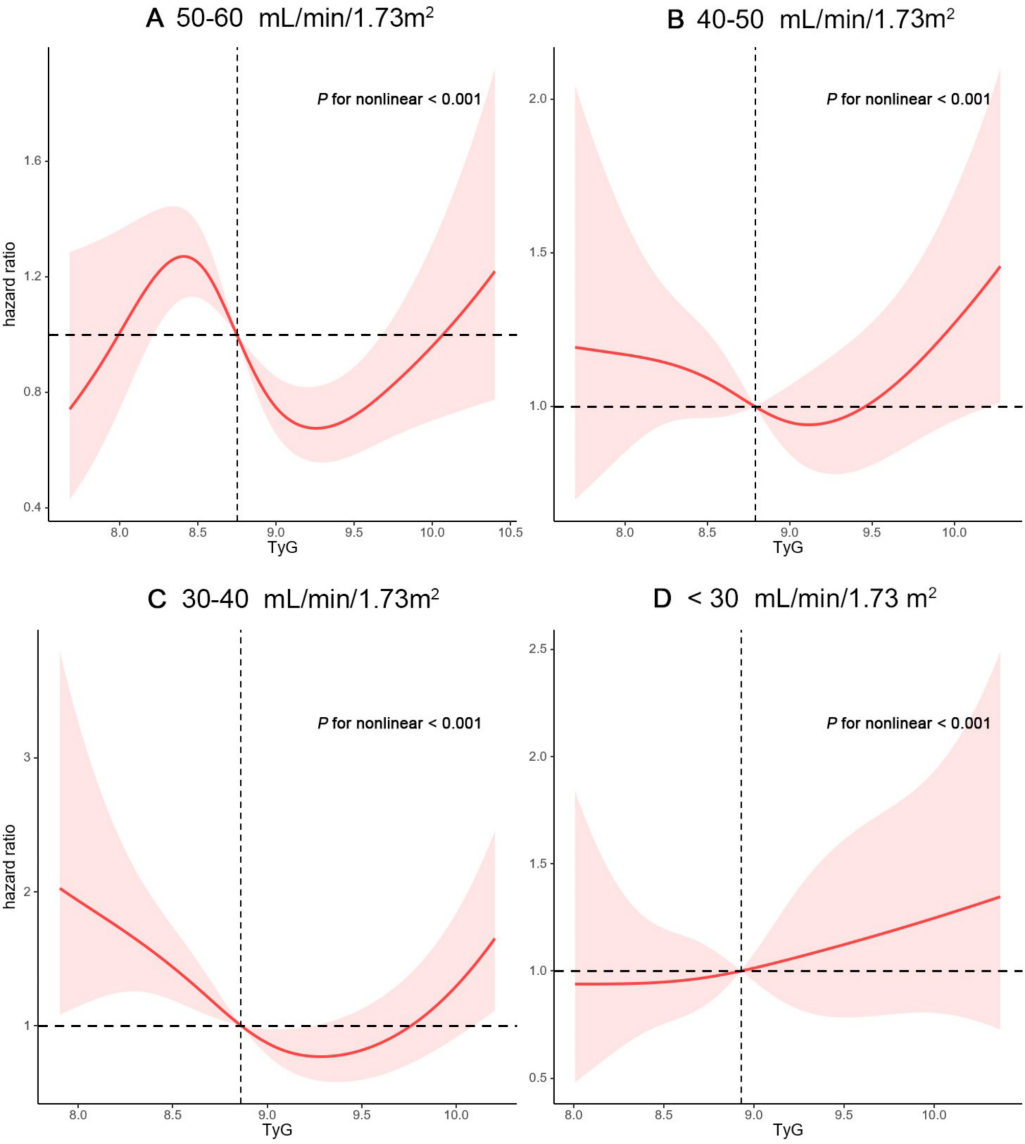
Hazard ratios for all-cause mortality based on restricted cubic spine functions for the TyG index in people with different renal insufficiencies (**A**, eGFR 50–60 mL/min/1.73 m^2^; **B**, eGFR 40–50 mL/min/1.73 m^2^; **C**, eGFR 30–40 mL/min/1.73 m^2^; **D**, eGFR < 30 mL/min/1.73 m^2^)

In subgroups stratified by eGFR, a nonlinear correlation was observed between TyG and death, with a sinusoidal association between the TyG and mortality in participants with eGFR levels of 50–60 mL/min/1.73m^2^ (*P* for nonlinearity < 0.001; Fig. 5A). In those with eGFR levels of 40–50 mL/min/1.73m^2^, the nonlinear demonstrated an approximately U-shaped relationship (*P* for nonlinearity < 0.001; Fig. 5B). In participants with eGFR levels of 30–40 mL/min/1.73m^2^ too, a nonlinear relationship between them was observed, but with an inverse spoon-shaped pattern (*P* for nonlinearity < 0.001; Fig. 5C). Nonetheless, in participants with eGFR < 30 mL/min/1.73m^2^, a monotonically increasing relationship was observed between them (*P* for nonlinearity < 0.001; Fig. 5D).

## Discussion

This retrospective analysis demonstrated that the correlation between all-cause mortality and the a high TyG index was not straightforward and that this association was affected by fluctuations in kidney function.

A range of clinical conditions, such as prediabetes, lipodystrophy, polycystic ovary syndrome, and nonalcoholic fatty liver disease, especially pertaining to metabolic syndrome and obesity, are associated with IR [16–19]. The TyG index is an economically viable and readily available indicator and can hence be used as a surrogate for IR. Liu et al. stated that an elevated TyG index, which is indicative of pronounced IR, is nonlinearly related to all-cause mortality [20], which agrees with the findings of the present study. A recent meta-analysis was unable to establish an independent link between the TyG index and death [10]. Nonetheless, such associations were influenced by factors such as diet, aging, and low BMI. As most studies have calculated the TyG index which based on the parameters that were measured during fasting in healthy individuals [4], it may be affected by hyperlipidemia or DM, which makes it challenging to exclude these confounders [21]. These observations support the idea that the application of this index is subject to variations among different populations. In the current study, in healthy individuals, the TyG index exhibited an optimal value in accordance with its original definition.

IR and renal insufficiency are tightly interwoven and potentially interact with each other via diverse mechanisms [14]. Moreover, both high TyG index and renal insufficiency have been reported to be risk factors for CVD and mortality [22, 23]. Several studies from China have observed that an elevated risk of CKD was associated with a high TyG index [24, 25], a claim supported by a recent meta-analysis [26]. Thus, the confluence of these risk factors should exacerbate the mortality risk. However, this hypothesis has not been tested previously, and the present investigation fills this research gap. This study examined the effect of renal function as a combined variable on the TyG index in two main ways. The findings revealed that the combination of a high TyG index and normal renal function incrementally increased all-cause mortality risk.

Furthermore, this survey found another intriguing result. The combination of a low TyG index and renal insufficiency exerted the greatest incremental effect on all-cause mortality risk, and a comprehensive evaluation indicated that renal insufficiency modifies the nonlinear relationship between the TyG index and death. The discovery significantly furthers the development of the TyG index in studies on adverse prognosis and may explain why its utility varies across studies. Moreover, the integration of the TyG index with renal function could augment its clinical utility considerably. Clinicians usually do not stratify patients when attempting to determine the prognosis and merely rely on findings from simple population-wide studies. This approach might lead to the misclassification of high-risk groups owing to the differential effects of the TyG index in individuals with renal insufficiency. Previous research had examined the combined effect of the TyG index with systolic blood pressure and BMI on all-cause mortality [13, 27]. Nevertheless, studies on the role and its optimal targets of TyG in different subpopulations are currently limited. Therefore, future research should delve into the application of IR across various subpopulations.

IR is often subclinical and is characterized by reduced insulin receptor levels in key metabolic tissues such as the liver, muscle, and adipose. This reduction leads to compromised insulin signaling, impairing glucose uptake at the cellular level. Emerging evidence indicates that insulin receptors are not only prevalent in the liver, muscle, and adipose tissues but are also notably present in the kidney [28, 29]. These receptors in the kidney play a crucial role in overseeing glucose homeostasis through various mechanisms [30–32]. Such discoveries highlight the kidney’s significant contribution to systemic glucose regulation. Moreover, the kidney is vital in modulating lipid metabolism in vivo [2], and a marked reduction in insulin receptor expression was noted in the cortex of the kidney of high-fat diet-fed rats and in patients with diabetes mellitus type 2 [33–36]. Consequently, individuals with renal insufficiency are susceptible to both glucose and lipid metabolic disorders, pushing the body towards insulin resistance. This shift not only influences the TyG index, derived from blood glucose and lipid levels but also heightens the risk of all-causes mortality. The exact impact of varying renal function on the TyG index’s ability to predict mortality risk remains unclear. It’s hypothesized that renal insufficiency might cause toxin buildup, inciting inflammation and oxidative stress [37], which in turn increases IR and affects the TyG index. Additionally, renal issues might alter lipid metabolism [2], further impacting the TyG index. Given the TyG index’s strong association with chronic disease risks, these alterations might jeopardize its predictive accuracy for mortality risk.

### Study strengths and limitations

The TyG index was considered to be a surrogate for IR in the current study to determine the impact of renal function on the ability of the index to predict all-cause mortality. The NHANES has laid down stringent regulations for assessing fasting lipid and glucose levels, which precludes the exclusion of confounding factors, such as hyperlipidemia and DM. Finally, to minimize the possibility of errors, the data were adjusted for actual and possible confounders.

There are certain limitations which are worth recognizing. Firstly, the current study collected the single relative index at the beginning, and this snapshot may not entirely reflect the long-term trajectory of the participants’ renal function and IR status. In addition, retrospective cohort design was used, and although multivariate risk regression modeling was performed to account for potential confounding factors, their influence on the results could not be completely ruled out. Moreover, samples with missing data were excluded, which might have introduced selection bias. Finally, despite using a nationally representative U.S. sample, the results may not be generalizable to other regions or individuals with specific diseases.

## Conclusion

It is well-known that higher TyG index is associated with higher all-cause mortality rates in community-dwelling adults. Taking into consideration that renal function could modulate the effect of the TyG index on all-cause mortality in this research, further work should involve investigation of the associations of renal function trajectories with the long-term cardiovascular outcomes.

### Electronic supplementary material

Below is the link to the electronic supplementary material.

**Supplementary Material 1: Sup Table 1**. The HR (95% CI) of cardiovascular and non- cardiovascular mortality according to TyG and different renal function from the three models

## Data Availability

The datasets used and/or analyzed during the current study are available from the corresponding author upon reasonable request.

## Abbreviations

CKD: Chronic kidney disease
CVD: Cardiovascular diseases
DM: Diabetes mellitus
FPG: Fasting plasma glucose
HDL-C: High-density lipoprotein cholesterol
IR: Insulin resistance
LDL-C: Low-density lipoprotein cholesterol
TG: Triglycerides
TyG: Triglyceride-glucose
