# Supplementary material for: Influence of renal function on the ability of TyG Index to predict all-cause mortality
Source: Lipids Health Dis. 2023 Nov 11;22:193. doi: 10.1186/s12944-023-01958-1 (PMC10638822; doi:10.1186/s12944-023-01958-1)
Supplement: Supplementary file 1 — Supplementary Material 1: Sup Table 1. The HR (95% CI) of cardiovascular and non- cardiovascular mortality according to TyG and different renal function from the three models [file 12944_2023_1958_MOESM1_ESM.docx]

**Sup Table 1. The HR (95% CI) of cardiovascular and non- cardiovascular mortality according to TyG and different renal function from the three models.**

| **Characteristics** | **Model 1** | |  | **Model 2** | |  | **Model 3** | |
| --- | --- | --- | --- | --- | --- | --- | --- | --- |
|  | **HR (95% CI)** | **P-value** |  | **HR (95% CI)** | **P-value** |  | **HR (95% CI)** | **P-value** |
| **Cardiovascular mortality** |  |  |  |  |  |  |  |  |
| TyG_L/eGFR_H | Reference | - |  | Reference | - |  | Reference | - |
| TyG_L/eGFR_L | 24.86 (18.53, 33.34) | <0.001 |  | 2.31 (1.80, 2.96) | <0.001 |  | 1.82 (1.38, 2.39) | <0.001 |
| TyG_H/eGFR_H | 2.2 (1.81, 2.66) | <0.001 |  | 1.41 (1.18, 1.69) | <0.001 |  | 1.12 (0.89, 1.42) | 0.330 |
| TyG_H/eGFR_L | 20.22 (15.97, 25.61) | <0.001 |  | 2.39 (1.88, 3.04) | <0.001 |  | 1.61 (1.20, 2.17) | 0.002 |
| **Non-cardiovascular mortality** |  |  |  |  |  |  |  |  |
| TyG_L/eGFR_H | Reference | - |  | Reference | - |  | Reference | - |
| TyG_L/eGFR_L | 13.11 (10.55, 16.29) | <0.001 |  | 1.74 (1.46, 2.08) | <0.001 |  | 1.50 (1.23, 1.83) | <0.001 |
| TyG_H/eGFR_H | 1.89 (1.65, 2.17) | <0.001 |  | 1.22 (1.07, 1.39) | 0.003 |  | 1.11 (0.96, 1.28) | 0.160 |
| TyG_H/eGFR_L | 10.50 (8.74, 12.61) | <0.001 |  | 1.63 (1.38, 1.93) | <0.001 |  | 1.33 (1.10, 1.61) | 0.003 |

Model 1: Not adjusted. Model 2: Adjusted for age, sex and race. Model 3: Adjusted for age, sex, race, education level, alcohol consumption, smoking status, BMI, LDL-C level, HDL-C level, eGFR, hypertension, DM and CVD.

CI, Confidence interval; TyG, Triglyceride-glucose index; HR, Hazard ratio.
